# The accuracy of fine-needle aspiration cytology for diagnosis of parotid gland masses: a clinicopathological study of 114 patients

**DOI:** 10.1590/1678-775720160214

**Published:** 2016

**Authors:** Jens Kristjan GUDMUNDSSON, Aida AJAN, Jahan ABTAHI

**Affiliations:** 1 - Linköping University Hospital, Department of Oral and Maxillofacial Surgery, Linköping, Sweden.; 2 - Eskilstuna Hospital, Department of Otorhinolaryngology, Eskilstuna, Sweden.

**Keywords:** Fine-needle, Citology, Parotid disease, Diagnosis, Biopsy

## Abstract

**Objective:**

Fine-needle aspiration cytology is a valuable method for preoperative assessment of head and neck tumors. However, its accuracy in detection of salivary gland masses is controversial compared with other methods. The aim of this work was to evaluate the effectiveness and accuracy of fine-needle aspiration cytology (FNAC) in the diagnosis of parotid gland masses.

**Material and Methods:**

Over a 10-year period, 126 parotid gland masses were resected. Retrospective chart reviews of 114 patients were performed. The results of FNAC and final histological diagnosis were compared and the accuracy of FNAC was determined.

**Results:**

Final histological evaluation revealed 11 malignant tumors and 103 benign lesions. Pleomorphic adenoma was the most common neoplasm (63%), followed by Warthin’s tumor (17.5%). The sensitivity of FNAC in detecting malignant tumors was 73% and the specificity was 97%. Positive predictive value (PPV) was 73% and negative predictive value (NPV) was 97%. The overall accuracy of FNAC in detecting parotid masses was 95%. False-negative diagnosis was found in mucoepidermoid carcinoma, acinic cell carcinoma, and epithelial-myoepithelial carcinoma whereas there was false-positive diagnosis in cases of pleomorphic adenoma and normal parotid gland tissue.

**Conclusion:**

FNAC is a reliable minimally invasive diagnostic method with a high sensitivity in diagnosis of lesions in parotid glands. The sensitivity of detection of malignant tumors in parotid glands was low due to the biopsy technique used, and depended on tumor location. Postoperative complications decreased after superficial parotidectomy.

## INTRODUCTION

Salivary gland tumors are a morphologically and clinically diverse group of neoplasms, which may present significant diagnostic and management challenges. In the western world, the estimated overall incidence is approximately 2.5‒3.0 cases *per* 100,000 *per* year^21^. Malignant salivary gland neoplasms account for more than 0.5% of all malignancies and approximately 3‒5% of all head and neck cancers^21^. In Sweden, the incidence of parotid gland tumors is 0.77 cases *per* 100,000 women and 1.16 cases *per* 100,000 men. In comparison, the overall incidence of salivary gland tumors during the same year was 0.99 in women and 1.49 in men^7^. Several imaging modalities have been used for diagnosis of parotid masses, including magnetic resonance imaging (MRI) and computed tomography (CT)^16^ ^,^ ^28^. MRI provides anatomical information about parotid tumor location and can be a useful tool to determine whether the mass is benign or malignant^16^. Fine-needle aspiration cytology (FNAC) is a globally accepted method in the preoperative evaluation of head and neck tumors. This method provides excellent distinction between benign and malignant parotid tumors, and has other advantages: it is inexpensive, easy to perform, relatively painless, and well tolerated^1^. The procedure is a cytodiagnostic method whereby a needle is used to acquire a sample of cells and microparticles of tissue for morphological analysis. Compared with histological results, a concordance of 86% has been observed, with a specificity of 98%, a sensitivity of 84%, and a diagnostic accuracy of 94%^1^. However, the value of FNAC for the diagnosis of parotid gland masses. has been questioned due to its low sensitivity regarding malignancy, variation in reported results, and the belief that most parotid masses require surgery in any case^17^ ^,^ ^20^. Tumors of the parotid gland can be removed by superficial parotidectomy and total parotidectomy^10^. Enucleation of parotid gland masses is not the first choice of treatment, and may increase the risk of recurrence^29^. Several complications have been associated with parotid gland surgery, such as facial nerve disturbance, Frey’s syndrome, and great auricular nerve paresthesia^9^ ^,^ ^27^. Factors that affect facial nerve dysfunction include malignant tumors, lesion sizes, operating time, and type of parotidectomy (total vs. superficial)^2^ ^,^ ^3^ ^,^ ^9^ ^,^ ^27^. The aim of this study was to evaluate the diagnostic value of FNAC in detection of parotid gland tumors at our clinic (especially for detection of malign lesions). The efficacy of FNAC was determined by comparing cytological results with final histological results in a retrospective study. In addition, clinical outcome of surgery and postoperative parameters were also evaluated.

## MATERIAL AND METHODS

### Identification of study population

Eskilstuna Hospital provides healthcare to more than a quarter of a million inhabitants. Over a 10-year period (January 2003 to December 2014), masses in the salivary gland tumors of 126 patients were resected. Patients treated between January and December 2006 were excluded due to an error in the patients’ electronic medical records. The medical records of 12 patients could not be located, so 114 patients (61 males and 53 females) with a mean age of 56.4 years (range 18-85 years) were included in this study for further investigation. Three surgeons performed the surgical procedures. Intraoperative facial nerve monitoring with a nerve stimulator was used in all cases. We used our department’s electronic database and clinical files to identify all patients who were diagnosed with a mass in the parotid gland. The ICD-10 codes D11.0, D11.9, C08.8, C08.9, and C07.9 were used to identify subjects from the database; this was followed by review of the medical records to confirm the diagnosis.

Information regarding patient demographics, FNAC results, and final histological diagnosis results was evaluated. The postoperative outcome, including postoperative infection and hematoma, facial nerve disturbance, and Frey’s syndrome was also registered. The preoperative FNAC results were compared with the final histological diagnosis from the surgical specimens. Data on age, gender, tumor type, and postoperative complications (facial nerve disturbance, postoperative infection, Frey’s syndrome, and hematoma) were collected from the clinical records. Facial nerve disturbance was classified into four categories: (1) none, (2) transient (0‒2 months), (3) less transient (2‒12 months), and (4) permanent. Patients with radiologically verified and FNAC-verified cysts in parotid glands were excluded. Patients with inconclusive FNAC results were also excluded.

### Fine-needle aspiration cytology technique

FNAC was performed using a 22-gauge needle attached to a 10-mL plastic syringe mounted on a syringe holder for a single hand grip. The aspirated material was deposited onto five clean glass slides without using local anesthesia. The number of aspirations varied between 2 and 3, depending on the accessibility of the tumor. FNAC air-dried smears were stained with Giemsa stain and the slides were then examined under a light microscope (Olympus Cx31, Nishi Shinjuku 2-chome, Tokyo, Japan), and results were reported according to the World Health Organization guidelines regarding parotid gland neoplasm.

All FNAC samples and tumor specimens were analyzed by the same laboratory (Unilabs Laboratoriemedicin at Mälarsjukhuset, diagnostikcentrum Eskilstuna, Sweden). Patients with FNAC-verified malignant tumors were referred to the head and neck cancer center at Örebro University Hospital for further cancer treatment.

The cytology laboratory provided three types of histological results: (1) benign aspiration cytology, (2) suspicious or malignant aspiration cytology, and (3) non-contributory aspiration cytology (indeterminate or inconclusive).

### Statistics

FNAC-based diagnoses were compared with diagnoses from histological examination of specimens. We calculated the sensitivity regarding the presence of malignancy (true positive/true positive + false negative), specificity regarding absence of malignancy (true negative/true negative + false positive), positive predictive value (PPV) (true positive/true positive + false positive), negative predictive value (NPV) (true negative/true negative + false negative), and accuracy of FNAC (true positive + true negative/total). Confidence intervals were computed at the 95% level. The FNAC and final histological diagnosis results are presented in terms of frequencies and percentages. Kappa value was also used to compare the agreement between the FNAC-based diagnosis and the biopsy-based diagnosis. Statistical analysis was carried out using SPSS (version 12.0).

## RESULTS

### Demographic and histological outcome

One hundred fourteen patients with parotid masses (61 males and 53 females) with a mean age of 56.4 years (range 18‒85 years) were included in the study. Mean duration of follow-up was 66 months (range 3‒132 months). The female-to-male ratio was approximately 1 to 1.1. Tumors were mainly located in the superficial parotid lobe (87 cases, 76%), but 27 tumors (24%) were in the deep parotid lobe. FNAC of the deep portion of parotid mass was conducted in 7 cases (6%), whereas in 20 cases (17.5%) this procedure was performed using ultrasound-guided biopsy.

Parotid tumors were resected via superficial parotidectomy (n=91; 79.8%). Total parotidectomy was performed in 18 cases with deep-lobe tumors (15.8%), and enucleation or superficial partial parotidectomy was performed in 2 cases (1.7%). Final histological evaluation revealed 11 malignant tumors (9.6%) and 103 benign lesions (90.3%). Pleomorphic adenoma was the most common neoplasm (72 cases; 63%), followed by Warthin’s tumor (20 cases; 17.5%).

### Accuracy of fine-needle aspiration cytology

FNAC was performed in 94 patients (4 tumors in deep portions and 90 tumors in superficial parotid glands). In 20 cases (21%) with tumors located in deep parts of parotid glands, FNAC was complemented with ultrasound. In 108 of 114 cases (94.7%), preoperative FNAC and final histopathological findings were conclusive. The overall accuracy of FNAC for parotid masses was estimated to be 95%. False-negative diagnoses were found in mucoepidermoid carcinoma (n=1; 1.4%), acinic cell carcinoma (n=1; 1.4%), and epithelial-myoepithelial carcinoma (n=1; 1.4%) whereas there were false-positive diagnoses in cases of pleomorphic adenoma (n=2; 2.7%) and in normal parotid tissue (n=1; 1.4%) (Table 1).

**Table 1:** The accuracy of fine-needle aspiration cytology (FNAC). Comparison of FNAC and final histological diagnosis (FHD)

Accuracy of FNAC vs. FHD	Location S/D	Method FNAC/UGB	Diagnosed as cancer (false positive)		Correctly diagnosed (as benign)		Total
FHD Benign	No.	No.	No.	%	No.	%	No.
Pleomorphic adenoma	6,4	8,25	2	2.7	72	97.3	74
Warthin’s tumor	19/1	19/1	0	0	20	100	100
Normal parotid tissue	2	3/0	1	1.4	2	66.6	20
Basal cell adenoma	2	2	0	0	3	100	3
Sialoadenitis	1/0	1/0	0	0	1	100	1
Benign oncocytoma	0/1	0/1	0	0	1	100	1
Lymphatic tissue	1/0	1/0	0	0	1	100	1
Total	89/14	92/11	3		100		103

	**Location S/D**	**Method FNAC/UGB**	**Diagnosed as benign (false negative)**		**Correctly diagnosed (as malignant)**		**Total**
**FHD Malignant**	**No.**	**No.**	**No.**	**%**	**No.**	**%**	**No.**

Malignified	0/2	0/2	0	0	2	100	2
pleomorphic adenoma							
Acinic cell carcinoma	0/1	1/0	1	1.4	0	0	1
Mucoepidermoid carcinoma	0,5	2	1	1.4	2	66.6	3
Lymphoma	1/0	1/0	0	0	1	100	1
Epithelial-myoepithelial	0/1	1/0	1	1.4	0	0	1
Carcinoma							
Adenocarcinoma	0/3	0/3	0	0	3	100	3
Total			3		8		11
All cases	2/9	5/6	6		108		114

FNAC, fine-needle aspiration cytology; FHD, final histological diagnosis; UGB, ultrasound-guided biopsy.

FNAC results did not correlate with histology in 6 specimens (6.4%), 3 of which were benign (3%) and 3 malignant (3%). Final histological evaluation showed 11 malignant tumors (8 tumors were diagnosed correctly by FNAC and 3 were classified as benign). Thus, FNAC failed to give a correct cytological result in 3 cases (27.3%). The summary estimates that the sensitivity and specificity were 73% (95% CI 39‒94) and 97% (95% CI 92‒99), respectively. The PPV was 73% and the NPV was 97% (Table 2). The kappa statistic for the degree of agreement between FNAC and histological results was 0.94.

**Table 2:** The reliability of fine-needle aspiration cytology (FNAC) and correlation with final histological diagnosis (FHD)

Reliability of FNAC				
Kappa	Value (%)	95% confidence interval		
		Lower limit	Upper limit	
Sensitivity	73%	39%	94%	0.947
Specificity	97%	92%	99%	
Positive predictive value	73%	39%	94%	
Negative predictive value	97%	92%	99%	

**Correlation between FNAC and FHD**				
**Final histological diagnosis, FHD**				
**Cytological diagnosis, FNAC**		**Malignant**	**Benign**	**Total**

Malignant		8 (TP)	3 (FP)	11
Benign		3 (FN)	100 (TN)	103
Total		11	103	114

FNAC, fine-needle aspiration cytology; FHD, final histological diagnosis; TP, true positive; TN, true negative; FP, false positive; FN, false negative.

In summary, the study showed low sensitivity for malignant tumors. However, regarding the location of tumors, these three false-negative cases were located in the deep portion of the parotid gland and they were diagnosed by FNAC without ultrasound-guided biopsy.

### Surgical outcome and postoperative complications

Tumors were mainly located in the superficial lobe (87 cases, 76%), but 27 (24%) were in the deep lobe of the parotid gland. The most common complication of parotid gland surgery was transient facial nerve palsy. Of 114 patients, 39 (34%) had a mild transient palsy, 9 (7.9%) had a transient palsy lasting more than 2 months, and only one patient (0.9%) had permanent facial nerve palsy at 1-year follow-up. Of 39 patients with short-term facial nerve weakness (lasting 0‒2 months), 21 (53.8%) had tumors located in the deep parotid lobe. Postoperative infection was found in 7 cases (5.8%). Nine patients (7.5%) had a recurrence of the tumor, 1 patient (0.8%) had hemorrhage, and 3 patients (2.5%) developed Frey’s syndrome.

## DISCUSSION

FNAC is a simple, inexpensive, and atraumatic method for preoperative assessment of tumors, lymph nodes, and other lesions in the head and neck. It is a safe procedure requiring minimal equipment, with a very low risk of cancer cell implantation^11^ ^,^ ^25^. Fine-needle aspiration of parotid glands may lead to hemorrhage, facial nerve injury, and fibrosis^5^. Infarction or metaplastic transformation of benign tumor has also been mentioned in the literature^8^. In this study, there were a few cases of infection (5.8%), hemorrhage (0.8%), and Frey’s syndrome (2.5%) as a result of FNAC of the parotid mass lesions. No long-term facial nerve dysfunction was observed 1 year after surgery.

Several imaging modalities have been used for diagnostics of parotid masses, including CT-scan, MRI, and ultrasound-guided core needle biopsy (USCB)^15^ ^,^ ^16^ ^,^ ^28^. CT-scan and MRI provide information about size and location of the tumor. However, comparing these two methods, CT-scan accurately assesses parotid lesions while MRI shows the relationship to adjacent structures better^16^ ^,^ ^28^. In a clinical study by Inohara, et al.^16^ (1993), the relative value of FNAC and MRI in relation to the differential diagnosis of benign and malignant parotid mass lesions was investigated. Eighty-one patients with parotid mass lesions (60 benign and 21 malignant) who had undergone FNAC and MRI preoperatively were retrospectively reviewed. The sensitivity, specificity, and accuracy of FNAC and MRI were 90%/95%/94% and 81%/92%/89%, respectively, and the combination of FNAC and MRI conferred no diagnostic advantage over either modality alone^14^. Haldar, et al.^13^ (2015) reported the largest series of patients with parotid neoplasm who underwent USCB. One hundred twenty specimens were analyzed with FNAC, 313 were analyzed with USCB, and 259 surgical specimens were analyzed from 397 patients. The sensitivity and specificity of FNAC were 70% and 89%, respectively, and the corresponding figures for USCB were 93% and 100%. Similar findings have been reported by others^15^.

**Figure 1:**
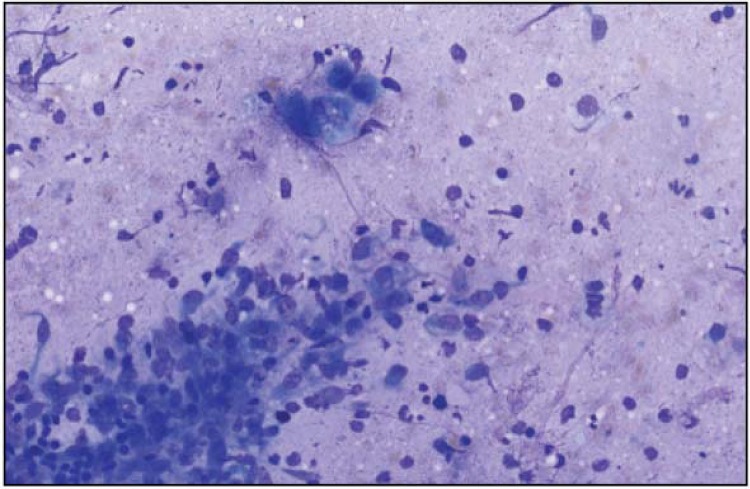
A low-grade mucoepidermoid carcinoma with thick mucinous material, intermediate cells, and only rare mitotic figures. Magnification: 100x

**Figure 2:**
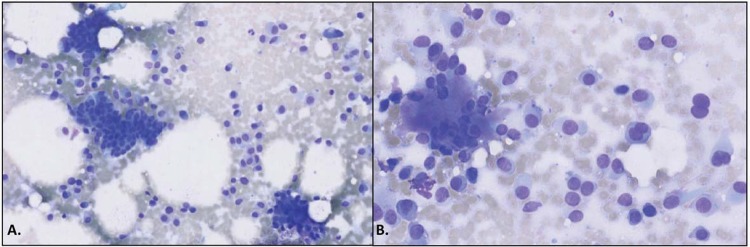
Patient with a pleomorphic adenoma with an epithelial cell component, a myoepithelial cell component, and a stromal (mesenchymal) component. Magnification: panel A, 100x; panel B, 200x

The importance of FNAC in distinguishing malignant parotid mass lesions from benign ones has been investigated by several authors^1^ ^,^ ^19^. Despite the simplicity of the method, the accuracy of FNAC varies depending on the precision and experience of pathologists^2^. Diagnostic difficulties with FNAC are most common in lesions involving pleomorphic adenoma, basal cell adenoma, low-grade mucoepidermoid carcinoma, and acinic cell carcinoma^3^. Moreover, the diagnosis is more difficult in low-grade mucoepidermoid carcinomas where thick mucinous liquid is seen in the background with a paucicellular smear^3^. In such cases, malignant tumors are mistakenly reported as benign lesions^3^. This occurred with one of our patients with a tumor in the deep portion of the parotid gland (Figure 1). The malignant tumor was assessed to be a benign tumor. Pleomorphic adenoma was one of the most common benign tumors in this study. Figure 2 shows cytological images of pleomorphic adenoma with its characteristic biphasic pattern, comprising epithelial/myoepithelial cells and fibromyxochondroid stroma.

In this study, of 103 cases of benign tumor, 100 cases were diagnosed using FNAC; of 11 malignant cases, 8 were diagnosed with FNAC. The overall accuracy of FNAC for parotid masses was estimated to be 95%, which is in the same range as in previous studies^4^ ^,^ ^12^ ^,^ ^22^ ^-^ ^24^ ^,^ ^30^. False-negative diagnosis was found in mucoepidermoid carcinoma (n=1; 1.4%), acinic cell carcinoma (n=1; 1.4%), and epithelial-myoepithelial carcinoma (n=1; 1.4%), and there was false-positive diagnosis in cases of pleomorphic adenoma (n=2; 2.7%) and in normal parotid tissue (n=1; 1.4%). Diagnosis of low-grade mucoepidermoid carcinoma is difficult due to its extreme morphological heterogeneity; it may be misdiagnosed as Warthin’s tumor or mucous retention cysts.

A review of the literature showed a FNAC sensitivity ranging from 54% to 92% and a specificity of 86‒100%^2^ ^,^ ^4^ ^,^ ^6^ ^,^ ^12^ ^,^ ^17^ ^,^ ^18^ ^,^ ^20^ ^,^ ^22^ ^-^ ^24^ ^,^ ^26^ ^,^ ^30^. These findings are summarized in Table 3. Parotid gland tumors account for approximately 3% of head and neck tumors and 80% of salivary gland tumors^7^ ^,^ ^21^. In this study, the incidence of pleomorphic adenoma and Warthin’s tumor was estimated to be 65% and 17.5%. FNAC was consistent with the final histological diagnosis in 98% of pleomorphic adenomas and in 100% of Warthin’s tumors.

**Table 3:** Sensitivity and specificity of fine-needle aspiration cytology (FNAC) for parotid gland tumors. Comparison with previous studies

Authors	Number	Sensitivity (%)	Specificity (%)
Orell^20^ (1995)	325	85.5	99.5
Al Khafaji, et al.^2^ (1998)	154	82	86
Stewart, et al.^26^ (2000)	341	92	100
Zbaren, et al.^30^ (2001)	228	64	95
Postema, et al.^22^ (2004)	388	88	99
Seethala, et al.^24^ (2005)	220	86	92
Aversa, et al.^4^ (2006)	310	83	100
Lin, et al.^18^ (2007)	279	63	97
Carrillo, et al.^6^ (2009)	135	92	98
Jafari, et al.^17^ (2009)	110	67	96
Schmidt, et al.^23^ (2011)	6169	80	97
Fakhry, et al.^12^ (2012)	202	80	89.5
Gudmundsson, et al. (2016) (this study)	114	73	97

## CONCLUSION

FNAC is a reliable method with high specificity for malignant parotid gland tumors, and provides the surgeon with valuable information in preoperative diagnostics. In this study, the sensitivity of FNAC in detection of malignant tumors was low due to the biopsy technique used, which depended on the location of the tumor. However, in cases where ultrasound guidance was used, 100% accuracy was achieved.
